# Resistance Exercise Participation in Community-Dwelling Older Adults in Korea: Associated Factors and Sex Differences

**DOI:** 10.3390/jcm13195900

**Published:** 2024-10-02

**Authors:** Seo Young Mun, Byung-Chan Choi, Jung Soo Lee, Yeo Hyung Kim

**Affiliations:** Department of Rehabilitation Medicine, College of Medicine, The Catholic University of Korea, Seoul 06591, Republic of Korea; symun2513@gmail.com (S.Y.M.); hjsrh@naver.com (B.-C.C.); drlee71@catholic.ac.kr (J.S.L.)

**Keywords:** aged, exercise, health risk behaviors, resistance training, sex

## Abstract

**Background/Objectives:** Despite the importance of resistance exercise in older adults, factors associated with participation remain unclear, especially regarding sex differences. This study investigated sociodemographic, behavioral, and comorbidity factors associated with insufficient resistance exercise participation among community-dwelling older adults in Korea, with analyses stratified by sex. **Methods:** Nationally representative cross-sectional data from 8671 participants aged ≥65 years (3758 men and 4913 women; mean age 72.8) were analyzed using the 6th-8th Korea National Health and Nutrition Examination Surveys (2014–2019). Complex-sample multivariable logistic regression identified factors associated with insufficient resistance exercise overall and by sex. **Results:** Women had a higher prevalence of insufficient resistance exercise than men (91.27% vs. 71.04%, *p* < 0.001). Older age groups, particularly those aged ≥80 years (OR: 2.39; 95% CI: 1.86–3.07), and those with lower education (OR: 1.56; 95% CI: 1.33–1.82), rural residence (OR: 1.89; 95% CI: 1.49–2.38), low household income, (OR: 1.45; 95% CI: 1.13–1.88), insufficient aerobic exercise (OR: 1.68; 95% CI: 1.46–1.94), current smoking (OR: 1.70; 95% CI: 1.26–2.29), and diabetes (OR: 1.28; 95% CI: 1.09–1.51) were independently associated with a higher likelihood of insufficient resistance exercise. The association of household income, current smoking, hypertension, and diabetes with resistance exercise adherence showed differences in sex-stratified analyses. **Conclusions:** Multiple sociodemographic, behavioral, and comorbid factors are linked to resistance exercise adherence among older Koreans. Tailored, sex-specific interventions are needed to promote resistance exercise in this population, informing public health strategies and further research on effective approaches for diverse older adults.

## 1. Introduction

Resistance exercise (RE) is a form of physical exercise that involves using resistance to induce muscular contraction, aimed at increasing strength, endurance, and muscle mass [1]. RE significantly influences various health aspects, including musculoskeletal health, cardiovascular well-being, metabolic function, overall mortality risk, and muscle strength development [2,3]. Its positive association with mental health, cognitive function, and quality of life emphasizes its broader significance compared with physical fitness [4]. Nevertheless, a decline in RE engagement has been observed with advancing age worldwide [5,6]. This decline is noteworthy, considering the role of RE in overall health maintenance in older populations.

Numerous studies have sought to elucidate the determinants of engagement in aerobic exercise and physical activity [7,8,9]. However, despite the well-documented health benefits of RE, relatively fewer studies have explored the factors associated with RE participation compared to aerobic exercise. The identified factors include age, gender, education, obesity, smoking, exercise-related decisional balance, perceived health status, self-efficacy, quality of life, and family social support [7,9,10,11,12,13,14]. Most existing studies, however, are limited by narrow inclusion criteria, such as focusing on specific age groups (e.g., individuals aged 36 years) [12], racial groups (e.g., African American and Caucasian populations) [10], or populations with specific health conditions such as cancer or ischemic heart disease [7,13,14]. For instance, a U.S. study found that age, body mass index (BMI), and cancer history were associated with adherence to RE guidelines among 1024 patients at average to high risk of cancer and cancer survivors [14]. Similarly, an association between adherence to aerobic exercise and RE was observed in a study of 531 Korean patients with ischemic heart disease [7]. Therefore, the current body of research remains insufficient for a comprehensive analysis of the factors associated with RE participation. Furthermore, there is a lack of research investigating the relationship between comorbidities and RE participation beyond demographic and behavioral factors.

Despite RE participation’s importance among older individuals, understanding the cofactors linked to RE participation in this population remains elusive. Although RE participation variability exists between the sexes [9,15], how it differs across the sexes remains unclear. Given the diverse health statuses and lifestyles of older adults, identifying the factors linked to RE participation is essential for developing targeted interventions and reducing social burdens by enhancing physical function. We hypothesized that factors such as socioeconomic status, lifestyle behaviors, and comorbidities would be significantly associated with RE participation and that these associations may vary between sexes. Therefore, our study aimed to uncover the sociodemographic, behavioral, and comorbidity factors associated with insufficient RE engagement among community-dwelling older adults using sex-stratified analyses.

## 2. Materials and Methods

### 2.1. Study Participants

This study used data from the Korea National Health and Nutrition Examination Surveys (KNHANES), a nationwide survey of health behaviors, chronic disease prevalence, and dietary intake among community-dwelling Koreans [16]. The KNHANES employs a stratified, multistage, clustered probability sampling method to collect a representative sample of Korea’s community-dwelling population. The KNHANES collects data through health interviews and examinations at mobile health centers, as well as nutritional surveys conducted during household visits [17]. KNHANES was performed every 3 years between 1998 and 2005 but has become annual since the 4th period (2007–2009). KNHANES data are de-identified to ensure individual privacy, and only anonymized raw data are accessible on the website (https://knhanes.kdca.go.kr (accessed on 3 July 2023).

The present study used data from the 6th, 7th, and 8th KNHANES between 2014 and 2019. Of the 47,309 participants during this period, this study included adults aged ≥65 years. Among the 9825 older adults (4240 men and 5585 women), 1154 individuals with missing data on RE participation were excluded. Ultimately, data from 8671 individuals (3758 men and 4913 women) aged ≥65 years were analyzed. Of the study participants, the unweighted prevalence of men and women was 43.3% and 56.7%, respectively. The mean age of the study population was 72.8 years (standard error [SE], 0.1). The KNHANES was approved by the Institutional Review Board of the Korea Centers for Disease Control and Prevention (IRB no. 2018-01-03-P-A and 2018-01-03-C-A), and informed consent was obtained from all participants. Participation in the KNHANES was voluntary, and participants were given small tokens of appreciation. This study was exempt from review by the Institutional Review Board of Uijeongbu St. Mary’s Hospital as it analyzed publicly available anonymized data.

### 2.2. Resistance Exercise

RE participation frequency was evaluated using the question: “In the past week, how many days did you engage in RE, such as push-ups, sit-ups, dumbbells, weights, or barbells?” Based on the World Health Organization (WHO) global recommendations on physical activity for health, engaging in RE for ≥2 days/week was categorized as sufficient participation [18]. Individuals performing RE less than two times a week were categorized as having insufficient RE. The participants’ aerobic physical activity and sedentary behavior were assessed using a validated Korean version of the WHO Global Physical Activity Questionnaire [19,20]. Participants were considered to have engaged in sufficient aerobic exercise if they participated in at least 150 min of moderate-intensity physical activity, at least 75 min of vigorous-intensity physical activity, or an equivalent amount of a combination of moderate- and vigorous-intensity physical activity [18]. Those who did not meet these recommendations were considered to have insufficient aerobic exercise. Participants with high sedentary time were those who accumulated ≥7 h of sedentary time per day, whereas those with low sedentary time were those with <7 h of sedentary time per day [21].

### 2.3. Covariates

The participants were categorized into 5-year age intervals (65–69, 70–74, 75–79, and ≥80 years). Participants’ education levels were classified as ≥12 years or <12 years. Place of residence was categorized as urban or rural, and household income was divided into quartiles (high, mid-high, mid-low, or low). The number of cohabitants was also recorded. Excessive alcohol consumption was defined as >20 g and >10 g of alcohol per day for men and women, respectively. Smoking habits were classified as never, past, or current smoking. Obesity status was categorized as underweight (BMI < 18.5 kg/m²), normal weight (18.5 kg/m² ≤ BMI < 23 kg/m²), overweight 23 kg/m² ≤ BMI < 25 kg/m²), and obese (BMI ≥ 25 kg/m²), according to the WHO Asia-Pacific guideline [22]. Participants with systolic blood pressure ≥140 mmHg or diastolic blood pressure ≥90 mmHg, diagnosed by a doctor, or taking antihypertensive medications were defined as having hypertension. Participants with a fasting blood glucose level ≥126 mg/dL or glycated hemoglobin level ≥6.5%, diagnosed by a doctor, or taking blood-sugar-lowering medications or insulin injections were classified as having diabetes. Participants with forced expiratory volume in 1 s/forced vital capacity (FVC) < 0.7, FVC < 80% of the predicted value on spirometry, or a diagnosis of chronic obstructive pulmonary disease or asthma were considered to have chronic lung disease. Participants with doctor-diagnosed myocardial infarction, angina, or stroke were classified as having cardiovascular disease. Participants diagnosed with their respective comorbidities by a doctor were defined as having cancer, arthritis, or depression.

### 2.4. Statistical Analysis

Since the KNHANES is a sample survey, it is essential to account for its complex survey design to ensure that the results are representative of the Korean population. Owing to unequal response probabilities and dropout rates in the sample data, the application of sampling weights corrects for biases caused by missing data, unequal sampling rates, and non-response errors. Therefore, all statistical analyses in this study were performed using weighted values derived from sampling weights, non-response-adjusted weights based on estimated response probabilities, and calibration weights that accounted for clustering and stratification within the survey data. Values are presented as weighted percentages (SE) for categorical variables and weighted means ± SE for continuous variables.

Characteristics of the men and women were compared using a complex-sample chi-square test. A multivariable-adjusted general linear model was employed to calculate the adjusted mean number of days per week of RE participation. We used a complex-sample chi-square test to compare the prevalence of individuals engaged in sufficient and insufficient RE among older adults. Multivariable-adjusted complex-sample logistic regression analyses were conducted to assess the association between sociodemographic, behavioral, and comorbidity factors and insufficient RE participation. In the logistic regression models, RE participation was categorized as sufficient (≥2 days/week) or insufficient (<2 days/week). Sociodemographic covariates included age group (65–69 years, 70–74 years, 75–79 years, or ≥80 years), educational level (≥12 years or <12 years), residential area (urban or rural), household income quartiles (high, mid-high, mid-low, or low), and presence of cohabitants (yes or no). Behavioral covariates comprised alcohol consumption (non-excessive or excessive), smoking habits (never, past, or current), aerobic exercise (sufficient or insufficient), sedentary time (low or high), and obesity status (underweight, normal weight, overweight, or obese). Comorbidity factors included the presence of hypertension, diabetes, cardiovascular disease, chronic lung disease, cancer, arthritis, or depression. Logistic regression analyses were performed for all older adults and separately for men and women to investigate these associations further. The results of the logistic regression analyses are presented as odds ratios (ORs) with 95% confidence intervals (CIs). Statistical Package for the Social Sciences version 22 (IBM SPSS Inc., Armonk, NY, USA) was used for analyses. Statistical significance was considered as *p* < 0.05.

## 3. Results

### 3.1. Participant Characteristics

Significant differences were observed between older men and women across various sociodemographic, behavioral, and comorbidity factors. Age distribution, educational level, household income, and the presence of cohabitants differed significantly between the sexes (Table 1). Behavioral factors, including alcohol consumption, smoking status, aerobic exercise adherence, sedentary behavior, and obesity status, differed significantly between the sexes (all *p* < 0.001), highlighting distinct behavioral patterns among older adults based on sex. Additionally, the prevalence of hypertension, cardiovascular disease (specifically diagnosed myocardial infarction, angina, or stroke), chronic lung disease, cancer, arthritis, and depression varied significantly between the sexes.

**Figure 1:**
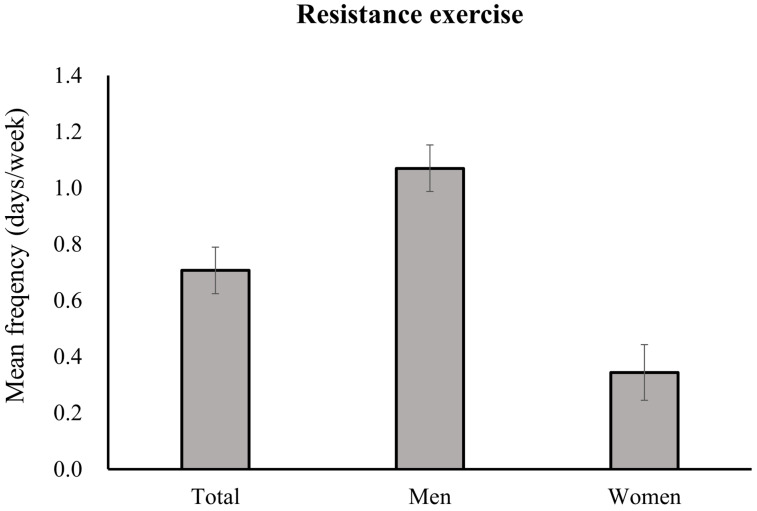
Adjusted mean weekly days performing resistance exercise in older adults according to sex. Values are adjusted for age, education, residence, household income, cohabitants, alcohol consumption, smoking, aerobic exercise, sedentary time, obesity, hypertension, diabetes, cardiovascular disease, chronic lung disease, cancer, arthritis, and depression. For total participants, values are additionally adjusted for sex.

**Figure 2:**
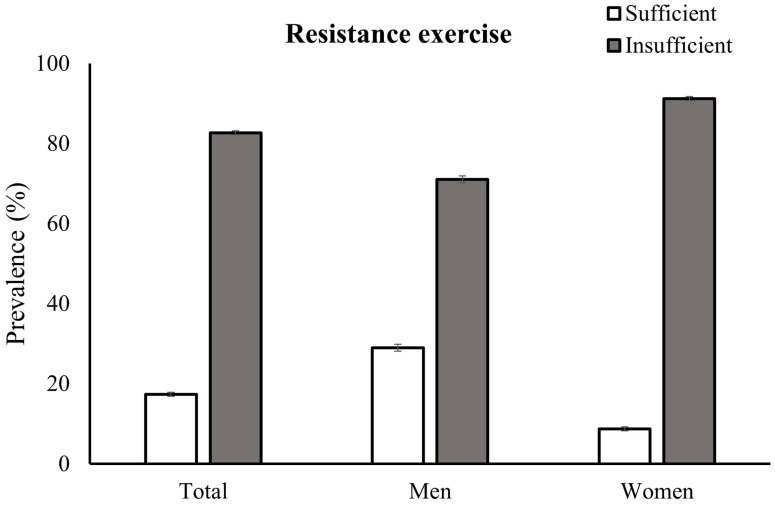
The weighted prevalences of resistance exercise adherence by sex.

The participants engaged in RE an average of 0.71 ± 0.08 days/week (Figure 1). Men had a higher mean adjusted RE frequency (1.07 ± 0.08 days/week) than women did (0.34 ± 0.10 days/week) (*p* < 0.001). Figure 2 illustrates the disparity in the prevalence of insufficient RE participation according to sex. Women had a significantly higher prevalence of insufficient RE engagement (91.27%; SE, 0.50%) than men did (71.04%; SE, 0.88%) (*p* < 0.001).

### 3.2. Factors Associated with Insufficient Participation in Resistance Exercise

Table 2 shows the sociodemographic, behavioral, and comorbidity factors associated with RE participation among older adults. Compared with those aged 65–69 years, significantly higher odds of insufficient RE participation were observed in older age groups, particularly those aged 75–79 years (OR: 1.53, 95% CI: 1.25–1.87) and ≥80 years (OR: 2.39, 95% CI: 1.86–3.07). Women showed higher odds of insufficient RE engagement than men did (adjusted OR: 3.84, 95% CI: 3.02–4.88). Older individuals with lower educational levels (<12 years) and a rural residence had a significantly higher likelihood of insufficient RE participation than did those with higher educational levels and urban residence. Individuals with low household incomes had significantly higher odds of insufficient RE engagement than those with higher household incomes.

Insufficient aerobic exercise participation was independently associated with higher odds of insufficient RE participation (OR: 1.68, 95% CI: 1.46–1.94). Current smokers exhibited a higher likelihood of insufficient RE engagement than never-smokers did (OR: 1.70, 95% CI: 1.26–2.29). The association of excessive alcohol consumption, past smoking status, high sedentary time, and underweight status with RE participation became insignificant after adjusting for potential confounders. Various comorbidities showed diverse associations with insufficient RE engagement before and after adjusting for confounders. After multivariable adjustment, diabetes retained its significant association with RE engagement (OR: 1.28, 95% CI: 1.09–1.51), whereas hypertension, chronic lung disease, and arthritis lost their associations with RE engagement. Cardiovascular disease, cancer, and depression were not associated with RE adherence in older adults.

### 3.3. Factors Associated with Insufficient Participation in Resistance Exercise by Sex

Table 3 presents the results of the sex-stratified analyses of the factors associated with insufficient RE engagement. The relationships between age group, education, residence, and cohabitant status with RE participation were consistent across the sexes. Both men and women in older age groups (75–79 years and ≥80 years) showed progressively higher odds of insufficient RE participation than the reference group (65–69 years) did. Lower education levels (<12 years) and rural residence were independently associated with insufficient RE participation in both sexes, with varying ORs. Low household income was significantly associated with insufficient RE adherence only in men.

A positive association was observed between aerobic exercise participation and RE among men (OR 1.70: 95% CI: 1.42–2.03) and women (OR 1.67: 95% CI: 1.29–2.16). However, an independent association between current smoking and insufficient RE engagement was identified in men (OR 1.84: 95% CI: 1.35–2.52); no association was found in women (OR 1.29: 95% CI: 0.52–3.22). No significant independent associations were observed between alcohol consumption, sedentary behavior, obesity status, and adherence to RE in both sexes.

Regarding comorbidity factors, sex-stratified analyses (Table 3) revealed differences, whereas non-stratified analyses did not (Table 2). Non-stratified analyses of the older population indicated that diabetes was the sole comorbidity independently associated with RE participation; however, this was only observed in men. Among women, the association between diabetes and RE participation became statistically insignificant after adjusting for confounding variables. The association between hypertension and RE participation varied between the sexes. Older men with hypertension had a decreased likelihood of insufficient RE participation (OR: 0.79, 95% CI: 0.65–0.97), whereas older women with hypertension had an increased likelihood (OR: 1.40, 95% CI: 1.09–1.79) of insufficient RE participation. Cardiovascular disease, chronic lung disease, cancer, arthritis, and depression were not associated with RE adherence in either sex.

## 4. Discussion

This study revealed that female sex, advanced age, low educational level, rural residence, current smoking status, insufficient aerobic exercise, and diabetes were independently associated with insufficient RE participation among community-dwelling older Korean adults. Additionally, the sex-stratified approach identified different associations between current smoking status, hypertension, diabetes, cancer, and depression with RE adherence between older men and women. Less than one-fifth of community-dwelling older adults engage in sufficient RE; community-based interventions are needed for public health promotion. This study’s findings can contribute to establishing tailored programs and sex-specific approaches.

The current study identified significant sex differences in RE adherence, with women being considerably less likely than men to engage in sufficient RE. The adjusted OR for insufficient RE participation among women relative to men was 3.75, the highest adjusted OR among various factors examined, suggesting that sex is a critical determinant of RE adherence in the Korean population. The impact of sex on RE participation has been controversial across previous studies. Our findings are consistent with those of previous studies suggesting that women are less likely to engage in RE compared with men [23,24,25,26]. Conversely, an Australian study involving adults aged 15–98 years reported that men were less likely to engage in RE than women [27]. This discrepancy highlights the potential influence of age and regional and cultural differences on sex disparities in RE participation.

The present study demonstrated a significant positive association between older age and insufficient RE participation in both sexes. Moreover, the ORs for the association between age and RE participation were the highest in the oldest age group. These findings align with those of previous studies indicating decreased RE adherence in older populations [24,25]. Our results may be related to the greater physical and motivational challenges in maintaining regular exercise routines among older adults. Numerous studies on older adults have highlighted a decline in muscle mass and strength as well as changes in body composition, resulting in reduced functional ability, flexibility, endurance, and physical activity [28,29]. Additionally, due to mental issues associated with aging, specific motivational barriers become more pronounced, further affecting participation in exercise [30].

Among various behavioral factors, aerobic exercise adherence was independently associated with RE adherence in the current study. According to previous large-scale studies in adults, the prevalence of adherence to RE, aerobic exercise, and both exercise guidelines was 9.9%, 30.2%, and 20.3% in the US [26] and 29.4%, 45.3%, and 22.6% in Germany [31], respectively. Our finding that 17.3% of older adults met the guidelines for RE is within the range reported in previous studies. Although previous studies have not focused on the association between participation in resistance and aerobic exercise, our results suggest that engagement in both types of exercise may be closely interconnected and clustered as part of positive health behaviors. Furthermore, aerobic exercise not only positively influences the reduction in comorbidities but also affects skeletal muscle and bone density [32,33]. Therefore, the benefits of aerobic exercise on body function may enhance RE participation, which can be challenging for older adults with comorbidities.

The present study found that current smoking was another behavioral factor significantly associated with insufficient RE participation. This aligns with previous research that indicated a negative association between smoking and RE participation [11]. The adverse health and motivational effects of smoking could contribute to this negative association as they impair physical performance and health-seeking behavior [34]. However, in our study, sex-stratified analyses revealed that current smoking was associated with a higher likelihood of insufficient RE engagement only in men. Smoking rates are consistently higher among men than women, especially in Asian populations [26,35], which may influence the sex disparity in RE adherence. Therefore, public interventions designed to enhance RE adherence among older adults who smoke should focus on current male smokers.

Regarding comorbidities, only diabetes was significantly associated with RE adherence in older adults, consistent with previous findings indicating a negative relationship between diabetes and physical activity levels [36]. However, the association between diabetes and insufficient RE participation was significant only in men. The higher cardiovascular morbidity and lower aerobic exercise capacity observed in women with diabetes may have contributed to the sex disparities in RE participation seen in our study [26,37]. Women with diabetes and low aerobic capacity may opt for RE as an alternative. Additionally, the association between diabetes and RE participation may be influenced by other sex-dependent demographic and socioeconomic factors, including advanced age, low income, and mental health issues [38]. Although the difference in OR between men and women was minimal, the wider 95% CI for women suggests that this sex difference may be attributable to greater variability in RE participation among women with diabetes or the relatively smaller sample size relative to the general population.

This study showed that the association between hypertension and participation in RE disappeared after adjusting for confounding variables. However, sex-stratified analyses revealed opposite associations: older men with hypertension showed a decreased likelihood of insufficient RE participation, whereas older women with hypertension exhibited an increased likelihood. Previous studies found that hypertension is negatively associated with physical activity levels and adherence to exercise guidelines [26,39]. However, studies analyzing this association in older adults by sex are lacking. Earlier studies reporting sex-specific attitudes and responses to chronic disease management partially support our findings [40]. Similarly, the sex discrepancy in our study may be related to the differences in health management and RE perceptions between men and women with hypertension.

In our study, educational attainment, residence, income, and cohabitant status were found to influence participation in RE, and these findings align with previous research [41,42,43]. Low educational attainment and rural residence were significantly associated with insufficient RE adherence. This association was consistently observed in analyses conducted separately for men and women and remained significant even after adjustment. Interestingly, as low-income individuals demonstrated an association with insufficient RE participation, this significance disappeared for women after adjustment. Additionally, the relationship between cohabitant status and RE participation initially appeared significant, but this association diminished after adjusting for potential confounders, regardless of sex. Overall, these findings highlight the complex interactions among sociodemographic factors that influence RE adherence, underscoring the need for tailored interventions that address these intertwined social and economic determinants of RE participation among older adults.

Previous studies have mostly been conducted in Western countries [23], and studies on RE-related factors among Asians are lacking. This large-scale study included a significant number of older adults and represented the entire community-dwelling older population in Korea. In addition, by stratifying by sex, differences between the sexes, not highlighted in previous studies analyzing the older population, were identified. This study’s results underscore the importance of considering sex disparities when promoting RE.

However, this study has limitations. First, it used cross-sectional data from the KNHANES, limiting its ability to infer causality. Second, this study relied on self-reported data, which may have been subject to recall bias and reporting inaccuracies. Additionally, because of the pre-defined variables and participant numbers in KNHANES, the sample size could not be determined prior to analysis, which may limit the generalizability of our findings. Furthermore, the large sample size may increase the likelihood of detecting statistically significant differences that are not clinically relevant, so caution is needed when interpreting and applying the clinical implications of our results. The KNHANES applies top-coding to the age variable to ensure anonymity, meaning the exact age of participants aged 80 years and older is not available, and specific age range information cannot be obtained. The questionnaire did not survey RE type and intensity, which could provide more detailed insights into RE behavior among older adults. The present study did not account for the complexity of daily living tasks, occupational activities, or sedentary lifestyles. Thus, the simple criteria used for defining sedentary time and aerobic exercise may not accurately reflect participants’ true daily activities, potentially affecting the study’s outcomes. Finally, we analyzed comorbidities individually and did not consider the impact of multimorbidity on older adults. Future studies should adopt a longitudinal design and utilize objective measures of RE, as well as expand the assessment of physical activity to include structured exercise and overall daily movement.

## 5. Conclusions

Multiple sociodemographic, behavioral, and comorbidity factors influence RE participation. As the relationship between these factors and RE adherence varies by sex, considering sex differences when designing public health interventions related to RE participation is essential. This study’s results can be used in programs promoting RE for health improvement in older adults. Additionally, the diverse and sex-specific factors associated with RE adherence highlight the importance of considering multiple dimensions of behavior and health in promoting RE among older adults. Although this study provides valuable insights into the factors influencing RE adherence within the older Korean population, it is important to note that the findings may not be directly generalizable to other populations or regions. Future studies should include randomized controlled trials and prospective longitudinal studies to infer causality. Further studies should be conducted to determine the most beneficial RE type and intensity for older populations across different geographical locations and diverse demographic groups.

## Figures and Tables

**Table 1:** Characteristics of community-dwelling older adults.

Variable	Total Participants	Men	Women	*p* Value *
Unweighted number (*n*)	8671	3758	4913	
Weighted number (*n*)	6,323,508	2,689,087	3,634,421	
Sociodemographic factors				
Age group (%)				<0.001
65–69 years	33.20 (0.64)	36.94 (0.96)	30.44 (0.77)	
70–74 years	25.94 (0.54)	27.59 (0.81)	24.72 (0.70)	
75–79 years	24.16 (0.56)	21.51 (0.73)	26.12 (0.76)	
≥80 years	16.70 (0.53)	13.97 (0.67)	18.73 (0.71)	
Education (%)				<0.001
≥12 years	26.92 (0.76)	42.50 (1.06)	15.38 (0.74)	
<12 years	73.08 (0.76)	57.50 (1.06)	84.62 (0.74)	
Residence (%)				0.488
Urban	75.74 (1.57)	76.12 (1.71)	75.45 (1.59)	
Rural	24.26 (1.57)	23.88 (1.71)	24.55 (1.59)	
Household income (%)				<0.001
High	10.4 (0.6)	12.7 (0.7)	8.8 (0.6)	
Mid-high	15.7 (0.6)	17.6 (0.8)	14.4 (0.7)	
Mid-low	27.0 (0.7)	30.1 (0.9)	24.7 (0.7)	
Low	46.8 (0.9)	39.6 (1.0)	52.2 (1.0)	
Cohabitants (%)				<0.001
Yes	79.7 (0.6)	89.0 (0.6)	72.8 (0.8)	
No	20.3 (0.6)	11.0 (0.6)	27.2 (0.8)	
Behavioral factors				
Alcohol consumption (%)				<0.001
Non-excessive	93.83 (0.31)	88.08 (0.63)	98.12 (0.22)	
Excessive	6.17 (0.31)	11.92 (0.63)	1.88 (0.22)	
Smoking (%)				<0.001
Never	62.65 (0.57)	20.45 (0.76)	94.11 (0.42)	
Past	27.93 (0.52)	60.73 (0.92)	3.47 (0.30)	
Current	9.42 (0.39)	18.82 (0.76)	2.42 (0.28)	
Aerobic exercise (%)				<0.001
Sufficient	32.94 (0.67)	39.24 (0.96)	28.24 (0.82)	
Insufficient	67.06 (0.67)	60.76 (0.96)	71.76 (0.82)	
Sedentary time (%)				<0.001
Low	43.42 (0.76)	47.08 (1.02)	40.58 (0.92)	
High	56.58 (0.76)	52.92 (1.02)	59.42 (0.92)	
Obesity status (%)				<0.001
Underweight	2.94 (0.21)	3.61 (0.34)	2.44 (0.26)	
Normal weight	33.74 (0.62)	35.94 (0.90)	32.10 (0.82)	
Overweight	26.44 (0.57)	27.96 (0.88)	25.31 (0.74)	
Obese	36.88 (0.62)	32.48 (0.93)	40.15 (0.80)	
Presence of comorbidity (%)				
Hypertension	63.31 (0.63)	59.15 (0.93)	66.38 (0.83)	<0.001
Diabetes	28.01 (0.57)	28.83 (0.87)	27.41 (0.74)	0.207
Cardiovascular disease	13.51 (0.42)	15.93 (0.66)	11.72 (0.55)	<0.001
Chronic lung disease	30.19 (0.62)	41.86 (0.95)	21.56 (0.69)	<0.001
Cancer	9.96 (0.38)	11.27 (0.58)	8.99 (0.48)	0.002
Arthritis	32.15 (0.58)	13.45 (0.63)	45.99 (0.82)	<0.001
Depression	6.36 (0.30)	3.03 (0.34)	8.82 (0.45)	<0.001

Data are presented as weighted percentages (standard error). * *p* values comparing men and women. *n*, number.

**Table 2:** Factors associated with insufficient resistance exercise participation in older adults.

Variable	Unadjusted	Adjusted *
Sociodemographic factors		
Age groups		
65–69 years	Reference	Reference
70–74 years	1.22 (1.04–1.42)	1.04 (0.87–1.24)
75–79 years	2.14 (1.80–2.55)	1.53 (1.25–1.87)
≥80 years	3.69 (2.94–4.63)	2.39 (1.86–3.07)
Sex		
Men	Reference	Reference
Women	4.26 (3.70–4.92)	3.84 (3.02–4.88)
Education		
≥12 years	Reference	Reference
<12 years	3.00 (2.62–3.45)	1.56 (1.33–1.82)
Residence		
Urban	Reference	Reference
Rural	2.32 (1.85–2.92)	1.89 (1.49–2.38)
Household income		
High	Reference	Reference
Mid-high	1.18 (0.93–1.49)	1.02 (0.79–1.33)
Mid-low	1.49 (1.20–1.86)	1.10 (0.86–1.42)
Low	2.84 (2.27–3.54)	1.45 (1.13–1.88)
Cohabitants		
Yes	Reference	Reference
No	1.97 (1.67–2.33)	1.01 (0.83–1.23)
Behavioral factors		
Alcohol consumption		
Non-excessive	Reference	Reference
Excessive	0.61 (0.48–0.78)	1.07 (0.82–1.39)
Smoking		
Never	Reference	Reference
Past	0.31 (0.27–0.35)	0.91 (0.73–1.13)
Current	0.63 (0.49–0.80)	1.70 (1.26–2.29)
Aerobic exercise		
Sufficient	Reference	Reference
Insufficient	2.36 (2.07–2.68)	1.68 (1.46–1.94)
Sedentary time		
Low	Reference	Reference
High	1.28 (1.11–1.48)	1.04 (0.89–1.22)
Obesity status		
Underweight	1.66 (1.09–2.53)	1.32 (0.85–2.05)
Normal weight	Reference	Reference
Overweight	0.88 (0.74–1.03)	0.92 (0.77–1.10)
Obese	1.09 (0.92–1.28)	1.00 (0.84–1.19)
Presence of comorbidity **		
Hypertension	1.25 (1.09–1.44)	0.96 (0.82–1.13)
Diabetes	1.27 (1.10–1.47)	1.28 (1.09–1.51)
Cardiovascular disease	1.19 (0.98–1.43)	1.19 (0.96–1.46)
Chronic lung disease	0.67 (0.58–0.76)	0.97 (0.83–1.13)
Cancer	0.87 (0.71–1.07)	1.00 (0.79–1.26)
Arthritis	1.79 (1.54–2.07)	0.93 (0.78–1.11)
Depression	1.29 (0.96–1.73)	0.84 (0.60–1.18)

Data are presented as odds ratios (95% confidence interval). * Adjusted for age, sex, education, residence, household income, cohabitants, alcohol consumption, smoking, aerobic exercise, sedentary time, obesity, hypertension, diabetes, cardiovascular disease, chronic lung disease, cancer, arthritis, and depression. ** The reference group consists of individuals without the respective condition.

**Table 3:** Factors associated with insufficient resistance exercise level by sex.

Variable	Men		Women	
	Unadjusted	Adjusted *	Unadjusted	Adjusted *
Sociodemographic factors				
Age group (years)				
65–69	Reference	Reference	Reference	Reference
70–74	1.10 (0.91–1.34)	0.98 (0.79–1.22)	1.41 (1.09–1.84)	1.18 (0.89–1.56)
75–79	1.94 (1.55–2.42)	1.57 (1.22–2.02)	2.04 (1.49–2.79)	1.42 (1.01–2.01)
≥80	3.04 (2.30–4.01)	2.38 (1.77–3.19)	4.05 (2.64–6.21)	2.24 (1.42–3.54)
Education (years)				
≥12	Reference	Reference	Reference	Reference
<12	1.96 (1.66–2.32)	1.45 (1.20–1.75)	2.46 (1.89–3.22)	1.80 (1.32–2.44)
Residence				
Urban	Reference	Reference	Reference	Reference
Rural	2.64 (2.05–3.40)	2.09 (1.60–2.74)	2.01 (1.41–2.86)	1.52 (1.05–2.21)
Household income				
High	Reference	Reference	Reference	Reference
Mid-high	1.11 (0.83–1.48)	0.99 (0.73–1.35)	1.17 (0.76–1.80)	1.23 (0.80–1.90)
Mid-low	1.46 (1.12–1.90)	1.06 (0.79–1.43)	1.44 (0.97–2.15)	1.18 (0.77–1.81)
Low	2.69 (2.05–3.53)	1.60 (1.18–2.17)	2.03 (1.38–2.99)	1.07 (0.69–1.67)
Cohabitants				
Yes	Reference	Reference	Reference	Reference
No	1.36 (1.06–1.76)	0.99 (0.74–1.32)	1.44 (1.11–1.87)	1.06 (0.78–1.43)
Behavioral factors				
Alcohol				
Non-excessive	Reference	Reference	Reference	Reference
Excessive	1.05 (0.81–1.36)	1.16 (0.88–1.54)	0.83 (0.37–1.84)	0.75 (0.34–1.65)
Smoking				
Never	Reference	Reference	Reference	Reference
Past	0.94 (0.77–1.16)	0.97 (0.78–1.20)	0.65 (0.36–1.19)	0.61 (0.33–1.14)
Current	1.81 (1.34–2.43)	1.84 (1.35–2.52)	1.46 (0.61–3.46)	1.29 (0.52–3.22)
Aerobic exercise				
Sufficient	Reference	Reference	Reference	Reference
Insufficient	2.12 (1.80–2.50)	1.70 (1.42–2.03)	2.08 (1.62–2.66)	1.67 (1.29–2.16)
Sedentary time				
Low	Reference	Reference	Reference	Reference
High	1.08 (0.91–1.28)	1.00 (0.82–1.21)	1.44 (1.14–1.83)	1.16 (0.90–1.49)
Obesity status				
Underweight	1.92 (1.15–3.21)	1.33 (0.79–2.23)	1.64 (0.77–3.46)	1.14 (0.54–2.40)
Normal weight	Reference	Reference	Reference	Reference
Overweight	0.75 (0.61–0.92)	0.83 (0.66–1.03)	1.15 (0.85–1.55)	1.18 (0.85–1.66)
Obese	0.84 (0.68–1.02)	0.97 (0.78–1.22)	1.27 (0.98–1.65)	1.08 (0.83–1.41)
Presence of comorbidity **				
Hypertension	0.91 (0.77–1.08)	0.79 (0.65–0.97)	1.74 (1.36–2.21)	1.40 (1.09–1.79)
Diabetes	1.23 (1.03–1.48)	1.28 (1.05–1.56)	1.56 (1.17–2.09)	1.23 (0.90–1.68)
Cardiovascular disease	1.36 (1.09–1.70)	1.21 (0.95–1.55)	1.39 (0.93–2.08)	1.13 (0.74–1.74)
Chronic lung disease	0.87 (0.73–1.03)	0.96 (0.80–1.15)	1.04 (0.79–1.36)	1.00 (0.76–1.33
Cancer	1.15 (0.88–1.51)	1.15 (0.87–1.54)	0.64 (0.46–0.91)	0.72 (0.50–1.03)
Arthritis	1.12 (0.87–1.43)	1.00 (0.76–1.30)	0.96 (0.75–1.22)	0.82 (0.64–1.06)
Depression	1.33 (0.78–2.29)	1.24 (0.72–2.12)	0.69 (0.48–0.99)	0.69 (0.46–1.02)

Data are presented as odds ratios (95% confidence interval). * Adjusted for age, education, residence, household income, cohabitants, alcohol consumption, smoking, aerobic exercise, sedentary time, obesity, hypertension, diabetes, cardiovascular disease, chronic lung disease, cancer, arthritis, and depression. ** The reference group consists of individuals without the respective condition.

## Data Availability

The data presented in this study are openly available in [KNHANES] at https://knhanes.kdca.go.kr (accessed on 3 July 2023), reference number [16,17].
